# Liver Transplantation for Hepatic Metastases from Colorectal Cancer: Current Knowledge and Open Issues

**DOI:** 10.3390/cancers15020345

**Published:** 2023-01-05

**Authors:** Marianna Maspero, Carlo Sposito, Matteo Virdis, Davide Citterio, Filippo Pietrantonio, Sherrie Bhoori, Filiberto Belli, Vincenzo Mazzaferro

**Affiliations:** 1 General Surgery and Liver Transplantation Unit, Medical Oncology and Colo-Rectal Surgery, Fondazione IRCCS Istituto Nazionale Tumori, 20133 Milan, Italy; 2 Department of Oncology and Hemato-Oncology, University of Milan, 20133 Milan, Italy

**Keywords:** colorectal cancer, colorectal liver metastases, liver transplantation, transplant oncology

## Abstract

**Simple Summary:**

Over 40% of patients with colorectal cancer will develop liver metastases during the course of their disease. While resectable liver-limited colorectal liver metastases (CRLM) are potentially curable, prognosis is dismal for patients with unresectable disease. Liver transplantation can greatly improve survival in selected patients with unresectable CRLM. This article provides an overview of the current status of liver transplantation for CRLM, open issues and future directions for research.

**Abstract:**

More than 40% of patients with colorectal cancer present liver metastases (CRLM) during the course of their disease and up to 50% present with unresectable disease. Without surgical interventions, survival for patients treated with systemic therapies alone is dismal. In the past, liver transplantation (LT) for patients with unresectable CRLM failed to show any survival benefit due to poor selection, ineffective chemotherapeutic regimens, unbalanced immunosuppression and high perioperative mortality. Since then and for many years LT for CRLM was abandoned. The turning point occurred in 2013, when the results from the Secondary Cancer (SECA I) pilot study performed at Oslo University were published reporting a 60% 5-year overall survival after LT in patients with unresectable CRLM. These results effectively reignited the interest in LT as a potential therapy for CRLM, and several trials are undergoing. The aims of this article are to give a comprehensive overview of the available evidence on LT for CRLM, discuss the open issues in this rapidly evolving field, and highlight possible ways to address the future of this fascinating therapeutic alternative for selected patients with CRLM.

## 1. Introduction

More than 40% of patients with colorectal cancer present liver metastases (CRLM) during the course of their disease [1]. When feasible, radical liver resection represents the main treatment option and might offer long term survival [2,3,4]. While the diffusion of parenchymal-sparing surgical strategies has increased the number of patients amenable to resection [5,6], up to 50% of patients present with unresectable disease [7,8,9], and 5-year overall survival for patients treated with systemic therapies alone is less than 20% [10]. A significant proportion of patient with CRLM are oligometastatic, namely with the liver as the predominant or exclusive site of metastases. Recurrence after liver resection is often intrahepatic [11,12] and, despite repeat resections, the prognosis is mainly dictated by liver failure due to subsequent progression and relapse. In the early eighties, liver transplantation (LT) had been proposed as a potential curative option for patients with unresectable CRLM. Due to poor selection, ineffective chemotherapeutic regimens, unbalanced immunosuppression and high perioperative mortality, the results were dismal, with a high rate of early recurrences and a 5-year overall survival (OS) of 0–18% [13,14,15]. For the next thirty years, CRLM were no longer considered as an indication for LT.

The turning point occurred in 2013, when the results from the Secondary Cancer (SECA I) pilot study [16] performed at Oslo University Hospital were published reporting a 60% 5-year overall survival after LT in 21 patients with unresectable CRLM. These results effectively reignited the interest in LT as a potential therapy for CRLM. Over the last decade the Norwegian group further improved their results in the SECA II study [17] using more restrictive criteria, with several other Institutions starting their own trials on LT for CRLM.

**Figure 1:**
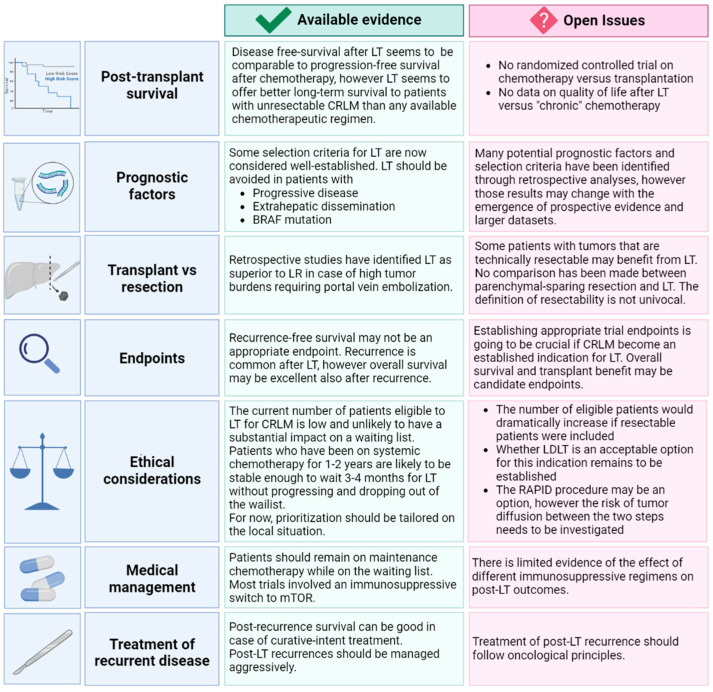
Overview of the available evidence and open issues in liver transplantation for colorectal liver metastases.

In this review, we will give a comprehensive overview of the available evidence on LT for CRLM (Figure 1). In addition, we will discuss the open issues in this rapidly evolving field and highlight possible ways to address them.

## 2. Survival after Liver Transplantation

Excluding case reports, the expected long-term outcomes of LT for CRLM in the modern era can be extrapolated from less than 100 cases. Published results come from the Norwegian experience [18] (SECA-I, SECA-II, and some results from the RAPID study [19], n = 56), a study from the Compagnons Hépato-Biliaires group [20] (n = 12) and the combined preliminary results of three trials on living-donor liver transplant from three North American centers [21] (n = 10). A number of other trials from Europe and North America are ongoing, most with anticipated ends within the next few years.

The best results in terms of overall survival were obtained in the SECA-II trial [17]: in this study the Norwegian group reported an OS of 83% at 5 years for 15 patients, with a median follow up of 36 months. This was obtained after refining the selection criteria of the SECA-I trial in which less restrictive entry criteria led to worst survival rates. In terms of survival duration, the cohort with the longest follow up is represented by the 23 patients of the SECA-I study [16,22] showing a 26.1% OS at 10 years with a median follow up of 58 months. Interestingly, when stratified by the Oslo criteria, the 6 patients who had more favourable disease presentation (Oslo score 0–1) showed a 10-year OS of 50%. Four patients of this cohort were alive and free from disease at a median time of 102 months, confirming that LT might truly cure a proportion of patients with unresectable liver metastases. Regarding living donor liver transplantation (LDLT), no data on long-term survival is currently available. Hernandez et al. [21] reported data on 10 patients who underwent LDLT at three North American Centers: with a median follow up of 1.5 years, the OS was 100%. Other forms of technically challenging LT for CRLM have been described: the RAPID procedure [19] (i.e., deceased or living transplant of a left lateral liver graft and ligation of the right portal vein, followed by hepatectomy of the native liver after sufficient regeneration of the graft) and the RAVAS technique [23] (heterotopic transplant of a left lateral liver graft into the splenic fossa after splenectomy, followed by total hepatectomy of the native liver after sufficient regeneration of the graft). The results of these procedures, that represent alternative strategies to increase the pool of accessible liver grafts, have only been described in case reports and data in terms of long-term survival cannot be extrapolated.

The alternative to LT for unresectable CRLM is palliative chemotherapy: to date, the only study comparing the two therapeutic options is a retrospective comparison of patients from the SECA-I and similar patients from the NORDIC VII [24] (a first-line chemotherapy study with FLOX chemotherapy scheme with or without cetuximab). In that study, Dueland et al. observed similar disease-free/progression-free survival (DFS/PFS: 8–10 months), even though the 5-year OS rate was 56% in patients undergoing LT compared with 9% in patients starting first-line chemotherapy. The reason for such a discrepancy between DFS/PFS and OS was mainly due to different metastatic patterns at relapse/progression, represented by slowly growing lung metastases in the LT group and progression of nonresectable LM in the chemotherapy group [24].

A randomized clinical trial (Transmet, NCT02597348) and two parallel studies (COLT, NCT03803436, and MELODIC, NCT04870879) are currently ongoing with the aim of comparing the long-term outcomes of LT with respect to different regimens of palliative chemotherapy in unresectable patients with CRLM. These trials have overall survival and not recurrence-free survival as their primary endpoint. Their results are expected in the next 2–3 years. A list of the currently active clinical trials on LT for CRLM is provided in Table 1.

### Open Issues in Survival after Liver Transplantation

As previously mentioned, very few prospective studies on LT for CRLM have been published during the last decade. Based on the presently available data and other non-randomized evidence, patients who undergo LT likely have longer overall survivals than those without. However, one must keep in mind that transplanted patients with CRLM were highly selected and would have been likely to have an outstanding prolonged survival on chemotherapy alone: outcomes of ongoing randomized and parallel studies comparing transplant versus chemotherapy are therefore needed to confirm the survival benefit of liver transplantation. On top of this, quality of life (QoL) after transplantation compared to “chronic” chemotherapy is still to be explored.

Transplantation is a life-altering event with significant associated short- and long-term morbidity and mortality and a need for lifelong immunosuppression. This could be considered an open issue of its own in the balance between risks and benefits of transplantation versus multiples lines of chemotherapy with or without liver resection and locoregional therapies. Nonetheless, post-transplant QoL in liver transplant recipients is reportedly higher than before transplant [25]. It increases over time and can eventually approximate that of the general population [26]. In addition, QoL has been found to be similar in patients undergoing transplantation or resection for hepatocellular carcinoma [27]. Conversely, “chronic” chemotherapy has a significant burden on the patient’s daily life, requiring frequent time-consuming hospital visits (the so-called time toxicity of systematic treatment [28]), as well as considerable side effects and a non-negligible risk of liver damage [29].

## 3. Prognostic Factors

The inclusion criteria of the SECA-I study were broad enough to allow an adequate patient accrual and assess the safety and feasibility of LT for CRLM according to modern transplant oncology principles. The only exclusion criteria were the presence of extra-hepatic disease and weight loss >10%. That study allowed the identification of tumor diameter >5.5 cm, pre-LT CEA > 80µg/L, time interval from resection of the primary tumor to LT <2 years and progression of the metastases during chemotherapy as the most significant predictors of worsened prognosis.

In the following SECA-II trial [17], the authors applied more restrictive selection criteria, as well as including patients with recurrent resectable CRLM. They enrolled 15 patients with at least 10% response to chemotherapy and an interval ≥1 year from diagnosis to transplant. The SECA-II patients had a significant lower number of metastatic lesions, size of the largest lesion, CEA levels, Fong Clinical Risk Score [30] and Oslo Score [31] than SECA-I patients and reported a 5-year OS of 83% with respect to the 60% of the SECA-I cohort. Median DFS in patients with less than 8 liver metastases on CT scan at time of LT was 24.3 versus 11.6 months in patients with more than 8 metastases (*p* = 0.083), and 24.3 months in node-negative tumors and 11.6 months in node positive ones (*p* = 0.065). Restrictive criteria therefore select patients with less aggressive biology; this, in turn, reflects into an improved prognosis.

Patients who were outside the aforementioned criteria were included in a separate arm of the trial (SECA-II arm D) and were transplanted using extended criteria donor grafts. This cohort also included patients with resectable or previously resected pulmonary metastases, or lack of response to chemotherapy, with the main exclusion criteria being BMI > 30 Kg/m^2^ and liver metastases larger than 10 cm. The long-term results of this cohort were quite dismal with median DFS and OS of 4 and 18 months, respectively. Interestingly, this trial showed that selecting patients with more favorable biological behavior (i.e., original SECA-II with respect to arm D) improves OS without a significant reduction in disease recurrence. In fact, DFS in the SECA-II arm D study and in the 14 patients with synchronous metastases of the original SECA-II study was similar, even though OS at 2 years was 100% in the SECA-II and 43% in the SECA-II arm D (*p* = 0.02).

**Figure 2:**
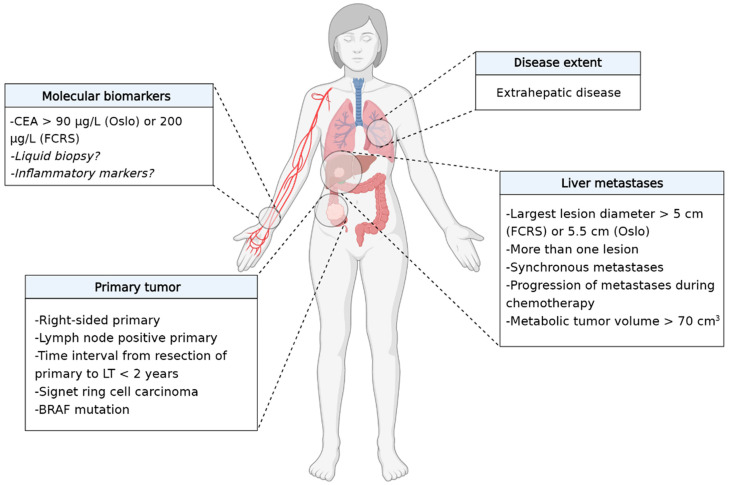
Established and potential risk factors associated with poor prognosis after liver transplantation for colorectal liver metastases.

Risk factors for poor prognosis after LT for CRLM are depicted in Figure 2.

### 3.1. Factors Associated with the Primary Tumor

Right-sided location of the primary tumor is a known poor prognostic factor for long-term survival, as seen in patients undergoing both liver resection [7,10,11] and palliative chemotherapy [12,13]: this was confirmed also in the LT setting, as evidenced by the results of SECA-II. Patients with right-sided primary tumors had a median DFS and OS of 3 and 12 months and all relapsed within 16 months after LT, while in left-sided tumors median DFS was 10 months and median OS was not reached (*p* = 0.104).

A key step for patients’ selection is an oncologic resection of the primary tumor that must be performed before LT. An interval ≥2 years from resection of the primary tumor to LT predicted DFS also in the Compagnons Hépato-Biliaires series [20]. Several histologic features of the primary tumor have also been found to predict prognosis: poorly differentiated and signet ring cell (mucinous) adenocarcinomas as well as BRAF mutation are associated with worsened survival [4].

In a study comparing selection criteria for LT based on a subpopulation of the SECA-I end SECA-II study, location of the primary tumor was further confirmed as a prognostic factor. In this study, PFS was 37 months in patients with right-sided tumors and 59 months in left-sided ones (*p* = 0.016) [31].

### 3.2. Metabolic Tumor Volume

Another important prognostic factor identified by the SECA trial is the metabolic tumor volume (MTV), measured from the pre-LT ^18^F-FDG-PET and defined as the tumor volume with ^18^F-FDG uptake segmented by a fixed threshold of 40% of the maximum standardized uptake value in the volume of interest. MTV was calculated as the sum of the values of each metastases [32]. Patients with low MTV (<70 cm^3^) had a significantly longer DFS, OS and PRS compared with patients with high MTV (>70 cm^3^). Five years OS for low and high MTV patients were 78% and 22%, respectively (*p* = 0.001) and 5 years RFS were 71% and 11%, respectively (*p* = 0.014).

### 3.3. Prognostic Scores

**Figure 3:**
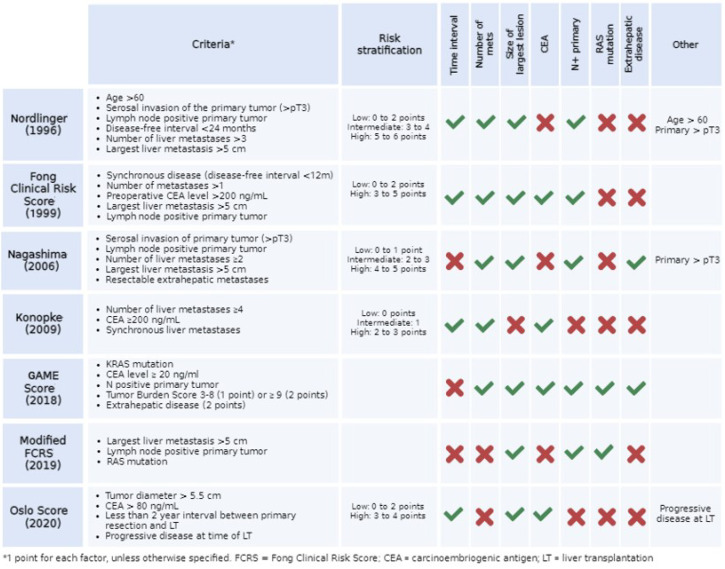
Prognostic scores for risk stratification of patients with colorectal liver metastases [20,21,23,25,26,27].

The main validated risk scores for patients with CRLM are summarized in Figure 3. Two of those have been applied to LT for CRLM and may be useful for risk stratification: the Fong Clinical Risk Score [30] (FCRS) and the Oslo score [31]. The FCRS was developed in 1999 for hepatic resection. FCRS ranges from 0 to 5; one point is assigned for each of the following: synchronous metastatic disease, lymph node positive primary, >1 lesion, size > 5 cm and CEA > 200 µg/L. In the study by Dueland et al. [31] comparing different selection criteria, a low FCRS (0 to 2) provided the most restrictive selection and the best long-term survival. Six out of 19 patients had a low FCRS and all of them were alive 33 to 147 months after LT, while 5-year OS in patients with high FCRS was 31% (*p* = 0.004).

The Oslo score, ranging from 0 to 4, is calculated by assigning one point for each of the following: largest lesion > 5.5 cm, CEA > 80 µg/L, time from surgery of the primary tumor to LT of less than 2 years. In the same series, the Oslo score was less restrictive but had a similarly good predictive power. Thirteen out of 19 patients had a low Oslo score (0 to 2) and their 5 years OS was 67%, compared to 17% of patients with a high Oslo score (*p* = 0.004).

### 3.4. Open Issues in Prognostic Factors

The available evidence on selection criteria is limited at best, as all available prognostic criteria have been derived retrospectively from univariate analyses. A summary of currently accepted and debated criteria for LT consideration in CRLM is provided in Table 2.

Some selection criteria have been proposed on a purely theoretical basis. For instance, a CEA ≤ 80 has been suggested as a selection criterion, but both in SECA I and SECA II the median CEA levels were below this threshold, 15 (1–2002) and 2 (1–30), respectively. We are far from understanding the true prognostic impact of pre-LT CEA levels, but it is likely that the CEA cut-offs for patient selection will be significantly lowered.

The interval from primary surgery to LT has been associated with post-transplant outcomes, with patients with more than 2 years from resection of the primary to LT showing more favourable outcomes both in the Norwegian and French experiences. This is the reflection of different favourable prognostic factors: (1) patients with metachronous metastases; (2) patients with synchronous liver metastases but with longstanding liver-limited disease due to either chemoresponsivity or oligometastatic presentation amenable to repeat surgical treatments. This is supported by the results of the study published by Ongaro et al. [43] in 2019 on determinants of extra-hepatic progression in patients with initially unresectable liver-limited CRLM, in which a higher number of liver metastases was associated with extrahepatic progression, while liver resections were protective. It is likely that, as more evidence is collected, different cut-off points will be established according to the different disease presentations.

The issue of biological non-resectability should also be considered. This is a concept applied to resectable CRLM in which, despite technical feasibility of the operation, liver resection has a high likelihood of resulting in potentially life-threatening morbidity and/or in early recurrence. Indeed, there are grey areas of CRLM presentations in which technical resectability is questioned. While parenchymal-sparing surgery has increased dramatically the resectability rate of patients with CRLM, not all patients with technically resectable disease are candidate for parenchymal-sparing approaches. Several studies have demonstrated that the higher the technical complexity, the higher is the risk of failure, particularly when advanced resective options such as ALLPS (Associating Liver Partition and Portal vein Ligation for Staged hepatectomy), or two-stage hepatectomy with portal vein embolization (PVE), venous deprivation, multi-step resections and other highly complex techniques are employed. As illustrated in Table 3, patients with risk factors for early recurrence due to presence of residual microscopic disease after resection, or who necessitate complex resection with considerable morbidity, might benefit from total hepatectomy and transplant. On the other hand, if the issue is unfavourable biology, then it is unlikely that transplantation will provide any benefit. The key point is that resectability should not be considered just as a technical issue for hepato-biliary surgeons.

The recently developed Genetic And Morphologic Evaluation (GAME) score [51] has not been applied to LT yet, but it might prove relevant as it incorporates a lower CEA cutoff (20 ng/mL), KRAS mutation, lymph node positive primary tumor, extrahepatic disease, and tumor burden score.

Finally, new diagnostic and prognostic markers are being investigated in the field of CRC, such as liquid biopsy. A liquid biopsy allows the assessment of either circulating tumor cells (CTC) or circulating tumor-derived DNA (ctDNA) in the bloodstream. The clinical utility of ctDNA and CTCs in metastatic CRC has not been completely understood, however it has been shown that change in ctDNA load after systemic treatment has a strong prognostic value [41]. In a recent trial by Osumi et al. [52], patients with lower ctDNA levels after 8 weeks of start of chemotherapy had significantly longer overall and disease-free survival than patients with higher ctDNA loads. A similar prediction of response to systemic therapy was observed by Tie et al. [42] in their analysis of 53 patients with metastatic CRC. ctDNA could become an important prognostic marker of therapy response in CRLM, as well as a tool for assessment of minimal residual disease after LT and for longitudinal surveillance of post-transplant recurrence.

In summary, selection criteria are at the heart of the current debate on LT for CRLM and are paramount to maximise the benefit of transplantation for this indication.

## 4. Liver Transplant vs. Liver Resection

It is well established that the best curative treatment for CRLM is an R0 surgical resection. Interestingly, LT for CRLM is debated more by its potential to offer a curative option to patients who are not amenable to surgery than as an alternative treatment for resectable disease. In fact, all currently published trials on LT for CRLM only include patients with unresectable disease, and no convincing evidence exists on LT for resectable patients.

The only ongoing trial on resection versus transplantation is the SECA II, whose Arm A includes patients with at least six technically resectable liver metastases randomized to either LT or LR. In addition, in the COLT trial (NCT03803436), patients can be included in case of technically resectable disease with a tumor burden score [44] >9, or in case R0 could not be achieved without complex parenchyma-regenerating procedures with a predicted risk of perioperative mortality > 3%, such as ALPPS (refer to the aforementioned concept of biologic non-resectability, and Table 2 and Table 3).

Resectability of liver metastases depend on factors related to patient conditions (i.e., patient resectability with known impact on morbidity and mortality after resection), to anatomical limitations (i.e., technical resectability, impacting on surgical feasibility of liver resection) and to tumor characteristics (i.e., oncologic resectability, impacting on long term outcomes after a R0 liver resection) [53].

It is self-evident that patients’ related factors influencing resectability would impact even more on transplantability, and thus it is unlikely that LT would become an alternative in such setting.

As mentioned, the definition of technical resectability is not straightforward and the place of LT in “borderline resectable tumors” would also be debatable. For instance, in a study by the Dutch Colorectal Cancer Group [54], inter-surgeon disagreement on resectability evaluation in CRLM was observed in 52% of cases, with major disagreement in 11% cases. Similarly, in a recent paper evaluating the agreement between 43 expert surgeons in selecting therapeutic options for patients suffering from CRLM, agreement on therapeutic strategies among them was none to minimal in more than half of the cases with kappa varying from 0.00 to 0.39 [55].

Finally, oncologic resectability is the setting where LT might enter as a novel therapy. In patients with high tumor loads and consequent poor outcomes after resection, LT could be an alternative option with significant impact on long-term survival. The only currently available comparisons between LT and LR derive from two retrospective studies. Dueland et al. [37] performed a retrospective analysis between patients with extensive CRLM who underwent LT versus PVE and liver resection. The advantage of LT was significant in patients bearing either low or high tumor burden. In patients with low tumor burden (defined as <9 nodules with maximum size < 5.5 cm) the 5-year OS of patients undergoing LT vs. PVE was 72.4% and 53.1%, respectively (*p* = 0.08), with worse tumor features being significantly more frequent in the LT population. In patients with high tumor burden, the median OS of patients undergoing LT was 40.5 months, significantly higher than the 19.7 months of patients undergoing PVE (*p* = 0.007). Similarly, Lanari et al. [36] retrospectively compared 128 LRs and 56 LTs performed at two Institutions. The two groups had similar long-term outcomes but significantly different oncologic characteristics at baseline. When selecting only patients with Oslo score ≤2 (optimal candidates for LT = favourable biology) and Tumor Burden Score (TBS) ≥9 (worst candidates for liver resection = high tumor burden), the 5-year OS after LT vs. LR was 69.1% vs. 14.6%, respectively (*p* = 0.002). As expected, the survival in patients with Oslo score >2 was similar between the two groups.

Similarly to that observed in LT for hepatic metastases from neuroendocrine tumors [33] LT and LR exhibit different patterns of recurrence, with recurrence more frequently occurring in the lung after LT and in the liver after LR. It can be argued that the high rate of intrahepatic post-resection recurrences is due to undetectable metastatic tumor foci left behind after LR that are effectively cleared with LT.

### Open Issues in Liver Transplant vs Liver Resection

The impressive survival outcomes achieved by the Oslo group in patients with low prognostic scores and low metabolic tumor volume may raise speculations that LT may improve survival in selected patients with resectable disease and favourable biologic prognostic features. For instance, in the paper by Lanari et al. [36], LT and LR display the same OS and DFS when the entire population is taken into consideration. In their study, a TBS > 9 plays a major role in determining a survival benefit after LT: patients with TBS > 9 might be considered for LT, while patients with low TBS are likely to have similar or better outcomes after resection.

As suggested by Dueland et al. [37], LT might represent an interesting alternative in patients who would otherwise require highly complex operations, such as ALPPS or two-stage hepatectomy with PVE. While the limitations of their retrospective study with a historical cohort should be considered, the potential implications for patients with extensive technically resectable disease must be recognized. However, can a 5-year OS of 33.4% be considered acceptable for the use of a limited resource such as a liver graft? In this same cohort, patients with unresectable, although low tumor burden had a 5-year OS of 72.4% after transplantation: a significantly higher survival than what is usually reported for CRLM after liver resection [56].

All this may raise the question on whether LT should be considered for selected patients with resectable CRLM and low tumor burden. Due to the restrictive selection process described above, CRLM patients currently represent only 1–2% of LT candidates [57], and that makes the indication to transplant for CRLM unlikely to generate a remarkable difference in current organ allocation. However, if patients with resectable disease were to become potential transplant candidates, the number of patients with CRLM might crowd the LT waitlist significantly. This would pose the ethical dilemma of organ resources being taken away from patients with established LT indications who have no other treatment options in favor of what might be only a marginal survival benefit in patients with resectable, liver-only CRLM. Such ethical considerations might be mitigated by the use of the RAPID technique [19] or living donors, however the latter also opens the question of donor safety in the face of a potentially limited survival benefit for the recipient.

## 5. Endpoints of Liver Transplantation

The appropriate endpoint of any LT for oncological indications should follow both oncological and ethical principles. While being cancer-free is certainly important to patients and to their quality of life, ultimately what determines a successful organ utilization are patient and graft survival [58]. Furthermore, the current evidence on LT for CRLM suggests that RFS might not be a reliable endpoint. The report by Dueland et al. [24] comparing patients from SECA I to patients in the NORDIC VII study [59] (first-line chemotherapy trial of Nordic FLOX +/− cetuximab) showed that RFS for LT was similar to progression-free survival for chemotherapy, although 5-year OS was 9% for chemotherapy and 56% for LT. As already mentioned, post-LT recurrence in these patients is not necessarily associated with decreased prognosis after LT. Of note, currently there are no reliable predictors of post-recurrence prognosis. In a study evaluating treatment of post-transplant recurrence [18], Fong score, metabolic tumor volume and Oslo score could not predict survival after the onset of palliative treatment, while they were associated with prolonged survival in patients undergoing curative treatment. The Oslo group also compared SECA patients [60] who underwent resection of post-transplant pulmonary metastases with a control group (non-transplanted rectal cancer patients with resected lung metastases), observing no differences in OS, DFS, and doubling time, thus implying that lung metastases after LT for CRLM behave as in a non-immunosuppressed setting.

### Open Issues in Endpoints of Liver Tansplant

LT is a drop in the ocean in the treatment of patients with unresectable CRLM but, as more robust evidence emerges, its use may gain traction in the oncological community, leading to an exponential increase in the number of potential candidates. During the time period in which the COLT trial accrued 22 candidates for LT, its parallel trial TRIPLETE (first-line chemotherapy randomized trial for unresectable CRLM) accrued 450 patients with similar inclusion criteria. The current prospective randomized (the Transmet study) and parallel trial (the COLT study) on LT vs. chemotherapy are likely to test the efficacy of LT in selected, oligo-metastatic, BRAF-negative CRLM vs. different chemotherapy regimens at incremental efficacy (i.e., conventional/triplet in TANSMET and triplet/quadruplet plus the anti–epidermal growth factor receptor antibody panitumumab in the COLT parallel trial).

If LT becomes an established and widespread treatment option for CRLM, comparative trials with appropriate endpoints should be undertaken. As mentioned, OS seems to be a more reliable outcome than RFS in this setting. However, OS requires a long-term follow-up and does not account for transplant-specific considerations. A possible alternative endpoint would be transplant benefit (TB) [61], namely the survival gain obtainable with LT compared to alternative non-transplant treatments. Differently from overall survival, TB takes into account urgency and utility, and may represent a more informative outcome for balanced organ allocation and patient prioritization in a setting in which intention-to-treat analyses are difficult to perform.

## 6. Ethical Considerations

One of the most debated issues when assessing a new indication to LT is how to manage its impact on the waiting list, particularly on the non-oncological indications to transplant. Clearly, this impact depends on two factors: the number of patients in need for a LT due to the new indication, and their need of prioritization to prevent their dropout from the list. With respect to the magnitude of the indication, it has been estimated that CRLM would account just for 1–2% additional patients to already accepted indications for LT, that is 0.24–0.51 patients per million people per year [57]. If correct, this relatively small patient population would hardly impact the outcomes of other waitlisted patients. This number would significantly increase if resectable patients were to be considered for LT as well. In terms of prioritization, CRLM should be treated like other oncological indications to LT and competition with high-MELD non-cancer patients as well as risk of waitlist mortality or disease progression should be considered. It is of note that if CRLM are listed after a mandatory interval of 1–2 years from primary tumor resection while responding to chemotherapy, the likelihood of remaining stable while waiting LT for 3–4 months (i.e., low risk of disease progression and drop-out) may be high. Consequently, although it is self-evident that waitlisted patients should be transplanted as soon as possible, it might be safe to assume that 3–4 months of median waiting time would be acceptable as well. Until more evidence emerges on predictors of drop-out or survival benefit of LT for CRLM, prioritization should be managed according to local scenarios.

### Open Issues in Ethical Considerations

Independently of the allocation system, the best tumor-related outcomes would be obtained with LT performed right at the time of listing. Patients in the two SECA trials had a median time on waiting list of less than 30 days, and a short time on waiting list is also expected for the TRANSMET trial, as all enrolled patients are granted an artificial MELD 40 at listing. It is unlikely that in the next future we will be able to guarantee these short waiting periods due to competition with patients with end-stage liver disease or other emerging urgent oncological indications, such as peri-hilar cholangiocarcinoma after neoadjuvant chemoradiotherapy, partial response to loco-regional therapies in hepatocellular carcinoma (HCC), and downstaging of HCC and cholangiocarcinoma after immunotherapy.

Whether longer waiting periods will translate in higher dropout rates due to progression or to worsened post-transplant outcomes is an important open question. In this setting, living donor liver transplantation has been proposed as a possible solution [21], particularly in those areas where organ availability would not allow for fast transplantation from deceased donors. As with any other LT indication, in LDLT the benefit to the recipient needs to be balanced with the potential risks to the donor. To date, only one study has been published reporting 10 patients who underwent LDLT for CRLM in three North American Centers. In addition, the resection and partial liver segment 2–3 transplantation with delayed total hepatectomy (RAPID) technique has been performed using grafts from both deceased and living donors with good outcomes [19,62]. Despite good perioperative outcomes, the long-term oncological outcomes of the RAPID technique are unclear, with concerns regarding leaving the tumour in situ in the presence of circulating hypertrophic growth factors during immunosuppression.

The utilization of deceased donors with “conventional” transplantation techniques could be greatly aided in the upcoming years by the use of dynamic organ preservation techniques and machine perfusion. Despite a certain degree of chemotherapy-associated steatohepatitis, the liver function of patients with CRLM listed for transplantation is usually better compared to that of patients listed for end-stage liver disease or HCC. This allows for the use of extended and even marginal grafts for these patients, especially if previously evaluated and reconditioned using machine perfusion strategies, both with normothermic and hypothermic machine perfusion. Indeed, normothermic machine perfusion allows the live evaluation of organs that would otherwise be discarded [63]. Hypothermic machine perfusion helps recondition the graft by decreasing the degree of ischemia reperfusion injury [64], with some reports even implying this might lower the risk of post-transplant cancer recurrence in the long term [65]. In addition, the emerging use of normothermic regional perfusion can certainly improve the utilization of organs from donation after cardiac death, for which patients with CRLM and good hepatic function would be ideal recipients [66,67].

## 7. Treatment of Recurrent Disease

LT for CRLM is associated with a high rate of recurrence: disease free survival at 1-year was 35% in SECA I (n = 25) [33], 53% in SECA II [17] (n = 15), 56% in the Compagnons Hépato-Biliaires study [20] (n = 12), 62% in the North American study [21] (n = 10). Differently from HCC [68], however, Dueland et al. has shown that recurrence after LT for CRLM does not seem to be predictive of overall survival and should not be used as a surrogate to evaluate outcomes of LT. Recently, the same Authors. [18] reported their outcomes of treatment of post-transplant recurrence in patients from the Norwegian experience, including patients from the SECA registry and RAPID study. Out of 56 transplanted patients, 44 had recurrence: all were able to receive treatment, of which 56% with curative intent, and 9 out of 44 have no evidence of disease at follow up. Five-year overall survival was higher after curative intent (51.3%, vs. 0% for palliative treatment, *p* < 0.001) and in patients with lower Fong score and MTV values. Most recurrences (52.3%) occurred in the lung, and pulmonary recurrences treated with curative intent had better 5-year post-recurrence survival (69.6%, vs. 25.4% for other sites, *p* = 0.02). A similar pattern of recurrence was observed also by the Compagnons Hépato-Biliaires [20].

Differently from the Norwegian experience, all 6 patients who had recurrence in the Compagnons Hépato-Biliaires study [20] were treated with either palliative chemotherapy or radiotherapy, and all had cancer-related deaths. In the North American cohort [21], 3 patients had recurrence, of which one in the peritoneum, one in the liver, and one outside the liver. All 3 were treated with palliative chemotherapy: one had a cancer-related death, while the other 2 have at least a 2-year survival without evidence of disease.

### Open Issues in Treatment of Recurrent Disease

A primary goal in LT for CRLM is to avoid recurrence. This can be attempted by optimizing selection criteria and immunosuppressive regimens. Adjuvant therapy may also be investigated, however it is not currently utilized after LT due to insufficient evidence of benefit [69] and concerns about graft loss.

All published series on LT for CRLM utilized mammalian target-of rapamycin (mTOR) inhibitors as maintenance immunosuppression. The immunosuppressive regimen for the SECA trials consisted of induction with basiliximab, followed by 4–6 weeks of tacrolimus and consequent conversion to sirolimus. Similarly, the North American cohort also performed induction with tacrolimus, steroids, and basiliximab followed by transition to mTOR inhibitors (either everolimus or sirolimus) approximately 6 months after LT. The Compagnons Hépato-Biliaires is the only cohort including patients (4/12, 33%) who were not switched to maintenance with mTOR, however there did not seem to be a difference in OS or RFS between the two immunosuppression groups.

In case of post-transplant recurrence, the available evidence suggests that it should be treated aggressively, especially if resectable, as per ILTS Transplant Oncology Consensus Conference recommendations [70].

While data from the Norwegian experience [18] and the preliminary contribution of North American cohort [21] show that patients may achieve remarkable survival and even cure after post-transplant recurrence, their treatment should not disregard state-of-the-art oncological practices. Poor-quality care must be avoided [71], including futile and excessive medical interventions, and treatments of dubious effectiveness that are based on provider urging instead of patient preference.

Much of the field of LT from CRLM still represents uncharted territory, and patients should be evaluated on an individual basis to ensure they get the best care, even if it implies the risk of a shift from cancer-directed therapy to symptom-directed palliative care.

## 8. Conclusions

Liver transplantation is a fascinating therapeutic option for patients with unresectable colorectal liver metastases, and may eventually be applied also to selected resectable patients. The current evidence is limited, but many trials are ongoing, and it is likely that this field will substantially grow during the next decade as experience increases and more knowledge becomes available on outcomes, selection criteria, and prognostic factors.

## Figures and Tables

**Table 1:** Currently active clinical trials on liver transplantation for colorectal liver metastases.

Clinical Trial Identifier Acronym	Timeline	Country	Interventions	Inclusion & Exclusion Criteria	Expected Enrollment
**Deceased donors, RCT**					
**SECAII** **NCT01479608**	2011–2027	Norway	LT vs. resection	- Arm A: resectable patients randomized 1:1 to LT vs. LR - CT for ≥3 cycles - If 2nd/3rd line CT, >10% RECIST response after CT - pN0 primary CRC - CEA <100 ng/mL at time of diagnosis of primary and metastases - ≤20 lesions, largest lesion ≤10 cm - Listing for LT >2 years after diagnosis of CRC	25
**TRANSMET NCT02597348**	2015–2027	France	LT vs. chemotherapy	- BRAF WT - Unresectable CRLM (validation committee) - ≥3 months tumor control after CT (SD or PR on RECIST) - ≤2 lines of CT - CEA < 80 ng/mL OR ≥50% decrease of the highest CEA	94
**SECAIII NCT03494946**	2016–2027	Norway	LT vs. chemotherapy	- Unresectable CRLM - Largest lesion ≤10 cm - ≥3 negative prognostic factors (CEA >80 ng/mL, <2 years from CRC diagnosis, largest lesion >55 mm - Previous resection of local recurrence or extrahepatic metastases <2 years OR resection of lung or hepatic hilum LN <1 year	30
**SOULMATE NCT04161092**	2020–2029	Sweden	LT vs. best alternative therapy	- Unresectable CRLM - No extrahepatic disease - At least 2 months of CT without progression - At least 1 year from initial CRC diagnosis - Liver metastases ≤ 10 cm - BRAF wild type - Microsatellite stable	45
**EXCALIBUR1** ** NCT04898504**	2021–2026	Norway	LT vs. HAI vs. standard of care	- Six or more liver metastases, planned for 2nd line chemotherapy - Extrahepatic disease or local recurrence treated at least 2 years before - Exclusion criteria - Arterial anatomy not suited for HAI - Previous bone or CNS metastatic disease - BRAF mutation - Any sign of extra-hepatic metastatic disease or local recurrence - Liver lesion > 10 cm - Age > 70	45
**Deceased donors, single arm, matched**					
**COLT NCT03803436**	2019–2024	Italy	LT vs. chemotherapy	- Unresectable CRLM with no extrahepatic spread - Primary pT3N1 max - RAS and BRAF wild-type - Sustained OR for at least 6 months, max 2 lines of CT - CEA < 50	25
**MELODIC NCT04870879**	2020–2025	Italy	LT vs. chemotherapy	- Unresectable CRLM (validation committee) - BRAF wild type - At least 3 months of CT - No extrahepatic disease or local recurrence - At least 10 months between primary resection and listing - CEA < 100 ng/mL - No lesion > 10 cm	18
**Deceased donors, single arm, non-matched**					
**SECAI NCT00294827**	2006–2023	Norway	LT		25
**TRASMETIR NCT04616495**	2021–2028	Spain	LT	- Unresectable CRLM - No extrahepatic disease - Response to ≤ 2 lines of CT - At least 1 year from CRC diagnosis to listing - BRAF wild type - Liver mets < 5 cm at latest imaging - CEA < 80 ng/ml	30
**NCT05185245**	2021–2030	Italy	LT	- Unresectable CLRM - Primary tumor *p* ≤ T4a - No local recurrence or extrahepatic disease - At least one CT line for at least 3 months with partial response or stable disease - CEA < 80 µg/L or reduction of ≥ 50% of highest CEA level	20
**NCT05398380**	2022–2026	Spain	LT	- Unresectable CRLM - No extrahepatic disease - Primary pT3N1 max - At least 2 months of CT, max 2 lines - At least 1 year from primary tumor resection to LT - -BRAF wild type - Liver mets < 5.5 cm at latest imaging - CEA < 80 ng/mL - ≥3 months tumor control after CT (SD or PR on RECIST)	35
**Living donor liver transplantation, single arm**					
**Toronto Protocol NCT02864485**	2016–2023	Canada	LDLT	- Primary CRC ≤T4a - Time from primary resection to LT ≥6 months - Unresectable CRLM with no macrovascular invasion - CT for ≥3 months with SD or PR - No prior liver resection/no recurrent metastases - BRAF wild type	20
**LIVERT(W)OHEAL NCT03488953**	2018–2023	Germany	LDLT	- Unresectable CRLM with no extrahepatic spread except resectable lung metastases - No PD after CT	40
**NCT05248581**	2019–2027	USA	LDLT	- Unresectable CRLM - CEA < 100 ng/dL	25
**NCT04874259**	2022–2026	Korea	LDLT	- Unresectable CRLM - No previous treatment - No extrahepatic disease	20
**NCT05175092**	2022–2030	USA	LDLT	- Unresectable CRLM (consensus of three HPB surgeons) - Primary max T3N2 - Oslo score 0–1 - At least 3 months CT with stable disease or partial response - No extrahepatic disease - BRAF wild type - Microsatellite stable	50
**LIVERMORE NCT05186116**	2022–2032	Italy	LDLT	- Unresectable CRLM - BRAF wild type - Primary tumor max pT3N1 - At least 4 months RECIST response to first line CT or disease control during second line CT - CEA stable or decreasing - No hereditary CRC - No prior extrahepatic disease or local relapse - No disease progression	25
**Resection and Partial Liver Segment 2/3 Transplantation With Delayed Total Hepatectomy**					
**NCT02215889**	2014–2028	Norway	RAPID	- Unresectable CRLM - ≥8 weeks of CT	20
**RAPID-Padova NCT04865471**	2020–2025	Italy	RAPID	- Unresectable CRLM (validation committee) - BRAF wild type - At least 3 months of CT - At least 6 months from primary resection and listing - At least 8 weeks of tumor control - No extrahepatic disease or local recurrence	18

LT = liver transplantation; LR = liver resection: RCT = randomized controlled trial; HAI = hepatic artery infusion; LDLT = living donor liver transplantation; RAPID = resection and partial liver segment 2–3 transplantation with delayed total hepatectomy; CRLM = colorectal liver metastases; CT = chemotherapy; PD = progressive disease; PR = partial response; SD = stable disease; CEA = carcinoembriogenic antigen.

**Table 2:** Currently accepted and debated criteria for liver Ttansplant consideration in case of colorectal liver metastases.

	Currently Accepted	Currently Debated
**Patient characteristics and setting**	Age ≤ 70	
	Performance status 0–1	Salvage transplantation in case of treatment-associated liver failure (e.g., hepatic artery infusion pump, chemotherapy-associated steatohepatitis, elective internal radiation therapy, post-intent-to-resection liver failure with/out untreatable vascular complication) [33] can be considered, although prognosis is worse than elective LT
**Primary tumor**	Left-sided	Some right-sided primary tumors with demonstrated favorable biology, as well as intraperitoneal rectal tumors, can be discussed as candidate for transplantation
	No nodal metastases	The impact of primary nodal metastases on prognosis is still to be investigated, as well as if N1 (N1a: 1 lymphnode vs. N1b: 2–3 lymphnodes vs. N1c areas of fat near the lymphnodes but not the lymphnodes themselves) [34,35] vs. N2 lead to different outcomes in the immonosuppressed contest of LT Presence of single, removable N+ at liver hilum at the time of transplant in a patient fulfilling all other transplant requirement may be considered for LT within investigational studies
	T stage < 4	The impact of primary T stage on prognosis is still to be investigated, especially whether T4a (invading the free serosa) vs. T4b (invading other organs/structures) lead to different outcomes in terms of local vs. systemic recurrences
	No signet ring cell histology	The impact of vascular/neural/immunotype of primary tumor on outcome is still to be investigated
**Disease extent**	No extrahepatic metastases	Patients with favorable biology after removal or sustained complete post-chemotherapy response or in case of resectable, limited pulmonary and peritoneal metastases are considered in some protocols of LDLT. Tumor infiltration by CRLM limited to the diaphragm may not be considered as a contraindication to LT.
**Response to medical and loco-regional therapies**	Stable and responding liver-only metastases to ≤2 lines of chemotherapy	Sustained tumor response after >2 lines of chemotherapy can be discussed as candidate for transplantation
	No current limits in number/size of hepatic metastases as long as the tumor is responding	Currently, no restrictions are applied for size and number of liver metastases, as long as response to chemotherapy is demonstrated. Size of the largest lesion >5.5 cm is associated with worse prognosis [31].
	Low metabolic tumor volume (MTV)	MTV of <70 cm^3^ measured at ^18^F-FDG-PET is associated with better patient outcomes [32].
**Hepatic tumor burden**	Unresectable disease	A trial testing the benefit of LT versus reseection in case of resectable CRLM is ongoing (SECA II Arm A, see Table 1). “Biologic non-resectable CRLM” can be considered for LT. Biologic non resectability can be inferred in: Patients with resectable liver-only disease but high tumor burden (TBS >9) lacking pan-RAS mutations [survival benefit of LT over resection has been observed in retrospective reports] [36] Patients candidate to advanced liver resection strategies considering remnant liver regenerative techniques with predicted mortality >10% (i.e., complex PVE, unfeasible second step of ALLPS); complex resections with liver inflow/outflow reconstructions [survival benefit of LT has been observed vs. PVE-associated resections in retrospective comparisons] [37] Patients with resectable and repeatedly recurring liver-only metastases (lacking pan-RAS mutations) after ≥3 curative hepatectomies performed in experienced Centers Comparison of LT with parenchimal-sparing R1par vs. R1vasc resection of CRLM [38,39] needs to be investigated
	Synchronous and metachronous metastases	No current limitation/stratification are applied with respect to the time from primary tumor to CRLM detection
	No BRAF mutation	Some specific molecular mutations [40] may be associated with better prognosis and may not be contraindications to transplantation k-RAS mutations are debated and not considered as contraindication in some studies
**Molecular characteristics**	CEA < 80 ng/mL	Various CEA cutoffs at the time of transplant. No current limitations with respect to CEA level at the time of first referral
**Biomarkers**	Circulating cancer byproducts (liquid biopsy)	ctDNA monitoring is increasingly utilized for decision making in CRC patients [41,42]
	At least 1 year between resection of the primary and transplant	At least 2 years between resection of the primary and transplant
**Timing**	At least 1 year between resection of the primary and transplant	At least 2 years between resection of the primary and transplant

ALPPS = associating liver partition and portal vein ligation for staged hepatectomy, LT = liver transplant, LDLT = living donor liver transplant, CRLM = colorectal liver metastasis, CEA = carcinoembryogenic antigen, ctDNA = circulating tumor cell DNA, PVE = portal vein embolization, R1 par = microscopic tumor infiltration involving liver parenchymal at pathology, R1 vasc = resection involving tumor detachment from the surface of a major intrahepatic vessel.

**Table 3:** Factors associated with biological unresectability.

Factor	Evidence	Rational for Transplantation
**Potential** **transplant benefit**		
Tumor bunder score (TBS) > 9 [44] Increasing number and size of metastases [45]	A TBS > 9 has been associated with reduced OS Increasing number (HR 1.3, 1.1–1.6) and diameter (HR 1.1, 1–1.2) associated with reduced OS.	Recurrence in the presence of these factor is likely to be due to microscopic, undetectable disease left behind during resection. The complete hepatectomy performed during LT may reduce this recurrence risk by eliminating all intrahepatic disease.
Need for intraoperative ablation [46]	Associated with early recurrence, OR 1.6 (1.1–2.5)	
Surgical margin 0 mm [46]	Associated with early recurrence, OR 1.5 (1–2.2)	
R1 resection [47] ^†^	Associated with early recurrence, HR 2.2 (1.2–4.2)	
Need for portal vein embolization [37,48]	Associated with reduced OS, HR 1.48 (1.09–1.98) In patients with high tumor load, median OS 19.2 months (95% CI, 0.0–39.5 months) after PVE vs. 40.5 months (95%CI, 26.3–54.7 months) after LT (*p* = 0.007)	
Initially unresectable disease [47]	Associated with early recurrence (HR 1.9, 1.02–3.7)	
More metastases detected intraoperatively [49]	Associated with reduced OS (HR 3.19, 1.28–7.97)	
Need for preoperative chemotherapy [45]	Associated with reduced OS, HR 1.7 (1.2–2.5)	
**Transplant** **benefit unlikely**		
More than 1 preoperative chemotherapy line [48]	Associated with early recurrence, RR 1.6 (1.1–2.4)	Recurrence in the presence of these factors is likely to be due to aggressive biological characteristics, thus a liver transplant is unlikely to change the prognosis
Progression during last-line chemotherapy [48]	Associated with early recurrence, RR 2.18 (1.11–4.47)	
Higher CEA levels [47]	CEA > 30 ng/m associated with early recurrence, HR 2.3 (1.2–4.7)	
Higher CA 19-9 [48] levels	CA 19-9 levels > 60 U/mL associated with early recurrence, RR 2.21 (1.44–3.43).CA 19-9 levels > 100 U/mL associated with reduces OS, HR 1.86 (1.37–2.48)	
Primary tumor T stage > 2 [45,46]	Associated with reduced OS, HR 1.4 (1.1–2) Associated with early recurrence, OR 2.6 (1.4–4.8)	
Right-sided primary tumor [45]	Associated with reduced OS, HR 1.5 (1–2.1)	
Primary tumor lymphovascular invasion [47]	Associated with early recurrence, HR 2.5, 1.3–4.8	
Nodal positive primary [48]	Associated with reduced OS, HR 1.46 (1.13–1.89)	

^†^ R1 refers to parenchymal R1, not vascular R1, which is not associated with increased recurrence rates [50]. PVE = portal vein embolization. TBS = tumor burden score. OS = overall survival. HR = hazard ratio. OR = odds ratio. RR = relative risk. CEA = carcinoembriogenic antigen. CA 19-9 = cancer antigen 19-9.
